# Does Avalanche Shovel Shape Affect Excavation Time: A Pilot Study

**DOI:** 10.3390/sports5020031

**Published:** 2017-05-23

**Authors:** Kurt Schindelwig, Alexander Hoffmann, Martin Mössner, Werner Nachbauer

**Affiliations:** 1 Department of Sport Science, University of Innsbruck, Fürstenweg 185, 6020 Innsbruck, Austria; alexander.hoffmann@student.i-med.ac.at (A.H.); werner.nachbauer@uibk.ac.at (W.N.); 2 Centre of Technology of Ski and Alpine Sport, University of Innsbruck, Fürstenweg 185, 6020 Innsbruck, Austria; martin.moessner@uibk.ac.at

**Keywords:** accident rescue, burial depth, rescue time, shovel rate

## Abstract

In Europe and North America, approximately 150 fatalities occur as a result of avalanches every year. However, it is unclear whether certain shovel shapes are more effective than others in snow removal during avalanche victim recovery. The objective was to determine the performance parameters with a developed standardized test using different shovel shapes and to determine sex-specific differences. Hence, several parameters were determined for clearing the snow from a snow filled box (15 men, 14 women). A flat (F) and a deep (D) shovel blade with the shaft connected straight (S) or in clearing mode (C) were used for the investigation of the shovel shapes FS, DC and the subsequent use of DC&DS. Mean snow mass shifted per unit time increased significantly from 1.50 kg/s with FS to 1.71 kg/s (14%) with DS and further to 1.79 kg/s (4%) with DC&DS for all participants. Snow mass shifted per unit time was 44% higher (*p* < 0.05) for men than for women. In excavation operations, the sex-specific physical performance should be taken into account. The results were limited to barely binding snow, because only with this snow did the tests show a high reliability.

## 1. Introduction

Every year, approximately 150 fatalities occur due to avalanches in Europe and North America [1], with most triggered by skiers and snowboarders [2]. One parameter for survival when a victim is critically buried (head under snow) in an avalanche is the average burial depth. In Switzerland, the average burial depth was found to be 0.7 m for survivors and 1.2 m for victims that died [3]. From 1980 to 2005, the median burial depth was 0.8 m in Switzerland and 1 m in Canada [4]. The primary cause of death among critically buried subjects was asphyxia (60%–86%), followed by trauma (9%) [5,6]. Hypothermia was of minor importance (1%) [7].

The probability of mortality increased with increasing duration of burial duration and burial depth in Austria and Switzerland. Compared to a burial duration of less than 15 min, the mortality was 18 times higher if the burial duration was between 36 and 60 min and 29 times higher if the burial duration was greater than 60 min. Mortality was almost five times higher if burial depth was greater than 120 cm compared to less than 40 cm [8]. In Canada, the survival rate showed an even earlier and steeper decline from 77% at 10 min to 7% at 35 min, which reflects a greater mortality resulting from trauma and an earlier onset of asphyxia, due to the denser snow [4]. This shows that there is only a very short time window for successful excavation in which rescue operations need to be as efficient as possible.

Rescue time includes the localization, as well as the excavation of the buried subject. Rescue time was reduced from an average of 29 min (1977–2000) to 18 min (2000–2006) mainly due to the improvement of transceiver technology [9]. Short rescue times are only possible if victims are rescued by their companions. This is also reflected in the probability of survival, which was 75% for victims rescued by their companions and 30% for victims rescued by an organized search team [10]. Only few data are available, which split the rescue time into localization and excavation time. Slotta-Bachmayr found that the excavation time takes about 30% of the rescue time [10], while Genswein and Eide reported that excavation requires the major part of the rescue time [11]. In contrast, rescue teams recovered the victim within about 90 min [10].

Rapid excavation can substantially reduce the overall rescue time and therefore mortality [8]. Edgerly and Atkins have shown the necessity for a strategic approach [9]. Genswein and Eide showed that minimal training increased the efficiency of companion rescue [11]. They have also developed a strategy for efficiently excavating buried persons called “the V-shaped snow conveyor belt” for several companions or rescue teams [12]. For this strategy, at least three rescuers work simultaneously in a V-shaped form, where the person at the tip cuts snow blocks and the other rescuers transport the snow with paddling motions to the back. The amount of snow which can be moved per scoop is a crucial factor in the excavation efficiency. Rescuers should ensure that snow does not glide off the shovel.

Rapid excavation requires rescue equipment to be carried. In South Tyrol, Italy, 80.6% of 4333 backcountry skiers carried standard rescue equipment (transceiver, avalanche probe and shovel). To reduce the risk of asphyxia, special safety equipment was developed: the air pocket. Victims buried for less than 15 min showed a survival rate of 95% using the air pocket compared to 69% without. In total, 67% of victims buried longer than 15 min survived using an air pocket compared to 4% without [8].

Another study investigated avalanche shovel models for defects regarding handling and durability [13,14]. Their recommendations for a good shovel comprised a blade with a serrated leading edge and a flat top, a telescope shaft and a D-shaped handle. Current avalanche shovels are made from aluminum, carbon fiber or plastics and vary in blade and shaft shape. Some offer the possibility of mounting the blade onto the shaft at a right angle (clearing mode).

Excavating a buried person is a physically strenuous task, which requires a great amount of strength and endurance. Working with maximum power quickly leads to fatigue. According to Laubach, women only have 60% of the upper body strength of men [15]. Therefore, in excavating a buried person, sex-related differences are expected.

A rescue operation involves freeing a buried person from snow as quickly as possible. Rapid excavation offers a good chance to increase survival among avalanche victims. Therefore, the objective of this project was to determine the time required for excavation, the snow volume per scoop, and the shoveling rate by using different avalanche shovel shapes; and to quantify sex-specific differences with a developed standardized test.

## 2. Materials and Methods

### 2.1. Test Arrangement

**Figure 1:**
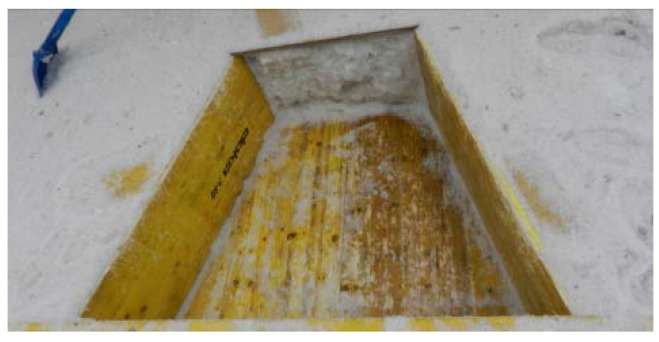
Empty box after excavation.

An open box was placed in the climate chamber (Kältepol, Natters, Austria) of the Centre of Technology of Ski and Alpine Sports, University of Innsbruck, Austria (Figure 1). The trapezoid-shaped box (parallel sides 0.75 and 1.45 m, height 1.10 m) had a depth of 0.5 m, resulting in a cross section of 1.21 m^2^ and a snow volume of 0.605 m^3^, in which a person lying in a squat position finds enough space. In 2008, Genswein and Eide proposed an alternating four-minute digging time for rescuers [12]. Therefore, a snow volume was chosen which could be removed in approximately the same time span. For each test, the box was completely filled with snow.

**Figure 2:**
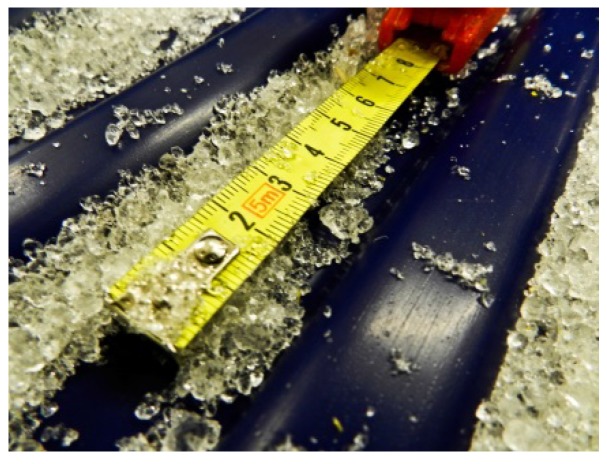
The sintered and granulated snow used for the tests.

Snow was collected from the surrounding area and transported to the climate chamber. There, the snow was left for several hours. Ice and snow particles bonded together, which is a process termed sintering by Kuroiwa [16] and Szabo and Schneebeli [17]. The snow was granulated by turning it over with shovels until the snow crystals had a size of 2–4 mm (Figure 2). Air temperature in the climate chamber ranged from −3.5 to −1.5 °C with humidity ranging from 29% to 33%, resulting in a snow temperature of about −2.5 °C. Throughout the investigation, nine times a snow volume of 0.02 m^3^ were weighed with a precision scale (DS 150K1, Kern & Sohn GmbH, Balingen, Germany). From these results, snow density was calculated.

### 2.2. Avalanche Shovel Shapes

**Figure 3:**
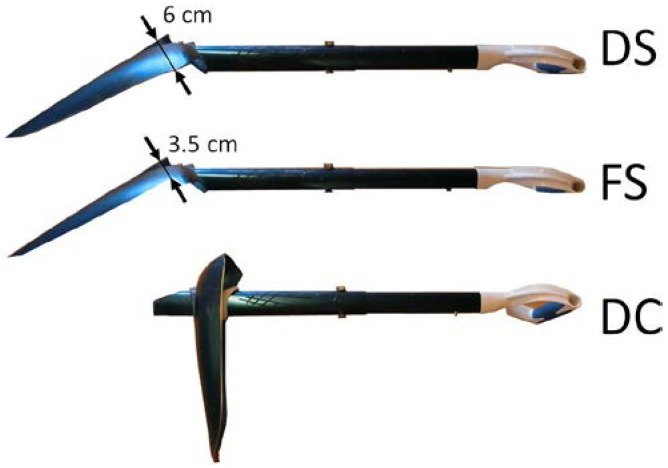
Deep shovel blade connected straight with the shaft (DS); flat shovel blade connected straight with the shaft (FS); deep shovel blade with the shaft connected in clearing mode (DC).

Three shovel shapes (Kodiak, Ortovox Sportartikel GmbH, Taufkirchen, Germany) (Figure 3) were tested: the shaft connected straight with the flat blade (FS); the shaft connected straight with the deep blade (DS); the shaft connected at a right angle (called clearing mode or hoe position) first and then with the shaft connected straight to the deep blade (DC&DS). The flat blade had a volume of 950 cm^3^ and the deep blade had a volume of 1400 cm^3^.

### 2.3. Study Participants

Fifteen men (26.3 ± 1.6 years, body mass index of 22.8 ± 1.6 kg/m^2^) and fourteen women (25.1 ± 3.8 years, body mass index of 20.9 ± 1.5 kg/m^2^) took part. All participants were healthy and exercised on a regular basis. The study was approved by the institutional review board of the University of Innsbruck.

### 2.4. Data Collection

Participants were instructed to clear the box of snow as quickly as possible. The box was considered clear when there was about 1 dm³ of snow in it. The same test leader always stopped the tests, if in his assessment this criterion was fulfilled. This task was performed six times in the sequence A–B–C–C–B–A, where A, B and C represent the randomly assigned shovel shapes. In the subsequent use of DC&DS, the participants were free to alternate from DC to DS as soon as they considered it more efficient. This shift was performed because the deeper the hole became the more inefficient the DC became. An extra DS shovel was at hand for shifting. All participants performed one excavation prior to the actual test in order to be familiar with the test requirements. The shoveling time was measured with a stopwatch. The excavation process was recorded with a video camera.

### 2.5. Data Analysis

The snow density (ρ) and the snow volume (V) being given, the mass of snow in the box (m) was calculated by m=ρV. The snow mass shifted per unit time (S) was calculated by S=m/t, whereby t refers to the time required for clearing the box. The number of scoops (A) was counted on the video recording. Hence, the snow mass per scoop (M) was given by M=m/A, the mean shoveling rate (f) by f=A/t and the snow volume per scoop ($V_{S}$) by $V_{S}=M/\rho$. The shoveling rate was determined for each ten-second interval, and the maximum shoveling rate was defined as the highest of these values.

All participants performed the test twice with each shovel shape. The results of both tests were averaged. Mean values and standard deviations (SD) were calculated for the entire group, as well as for male and female participants. Normal distribution was tested with the Shapiro–Wilk test. Differences between shovel shapes were analyzed by one-way analysis of variance (ANOVA for repeated measures) or Friedman tests in cases where the Shapiro–Wilk test showed that the assumption of normality could not be applied. Degrees of freedom were corrected by the Greenhouse–Geisser procedure, if Mauchly’s test indicated a violation of the assumption of sphericity (ANOVA only). Bonferroni adjusted paired-sample t-tests or Wilcoxon tests were used to follow up significant findings. These tests were independently performed for the subgroups of men and women, as well as for the entire group. Differences between women and men were analyzed via independent sample t-tests or Mann–Whitney U-tests, respectively [18].

## 3. Results

Snow density was 651 ± 8 kg/m^3^ during the tests resulting in a snow mass of 394 ± 5 kg for the 0.605 m^3^ box. Depending on the participant and the shovel shapes, the excavation took 3–5 min.

For both the 15 men and the 14 women, snow mass shifted per unit time increased significantly (14%, *p* < 0.001) from FS to DS. The increase from DS to DC&DS was not significant for either sex (men: 4%, *p* = 0.153; women: 4%, *p* = 0.092). For all participants, the increase of snow mass per unit time from DS to DC&DS was significant (4%, *p* = 0.011). Men moved a significantly (*p* < 0.001) larger snow mass per unit time than women (43% higher for FS, 44% for DS and 44% for DC&DS) (Table 1). The alternation from DC to DS occurred between 11 and 73% (31% ± 14%) of the test duration. 

The effective snow volume per scoop was 2.5–4 times larger (Table 1) than the volume of the shovel blades. For the entire group, the increase of snow volume per scoop from FS to DS (510 cm^3^, 15%, *p* < 0.001) and from DS to DC&DS (330 cm^3^, 6%, *p* < 0.001) was significant.

**Figure 4:**
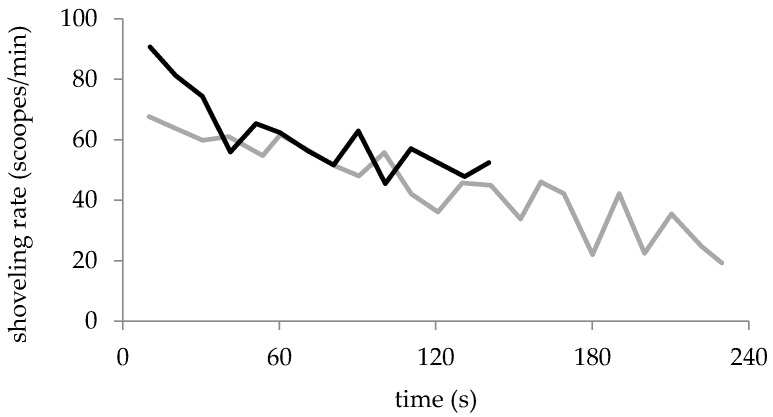
Shoveling rate versus time of a woman (grey line) and a man (black line) with DS. The shoveling rate was determined for each ten-second interval.

For men and women, the mean and maximum shoveling rates were not dependent on the shovel shapes (Table 2). The mean shoveling rate of men was significantly higher than that of women (26.6% higher for FS, *p* < 0.001; 26.3% for DS, *p* < 0.001 and 21.3% for DC&DS, *p* = 0.002). The maximum shoveling rate was significantly higher than the mean shoveling rate for men (89%, *p* < 0.001) and women (67%, *p* < 0.001). The maximum shoveling rate occurred in the first 20 s of the test. Figure 4 shows the shoveling rate versus time of a woman and a man using DS. The shoveling rate decreased over time.

## 4. Discussion

The objective of this investigation was to determine snow mass shifted per unit time, snow volume per scoop and shoveling rate for excavating a given amount of snow using three avalanche shovel shapes for women, men and the entire group with a developed standardized test. The results of the snow density measurements indicated that the snow consistency remained constant and allowed reliable tests.

The deep shovel blade (DS), with a 47% larger volume than that of the flat shovel blade (FS), both with a straight shaft, proved to be much more effective in the given test environment. Both the snow mass shifted per unit time and the snow volume per scoop were approximately 15% higher with DS as compared to FS. The increase in the snow volume removed per scoop corresponded to the difference in the volume size of the shovel blades. Interestingly, the mean and maximum shoveling rates did not depend on the shovel shape used. The smaller amount of snow removed per scoop with FS did not lead to an increase in the shoveling rate. The video recording showed that a considerable amount of snow on the shovel, in particular of FS, subsequently slipped off during the removal motion. This means that one intends to move the same amount of snow with FS and DS, but with FS a larger portion of the snow fell back into the box. As the snow fell back into the box, the excavation time increased accordingly. That means that reducing the snow mass per scoop of granulated snow when loading would improve the effectiveness of FS. Summarizing, for granulated snow, DS was most effective. However, for stronger binding snow, the effectiveness of FS may be equal to DS.

The subsequent use of DC&DS (deep blade in clearing mode, deep blade with a straight shaft) led to a further increase of 4% for the snow mass shifted per unit time and 6% for the snow volume per scoop. These differences were not significant. The participants alternated from DC to DS between 11% and 73% of the excavation time, which indicates that the preference for DC or DS among the participants varied considerably. If DC were used effectively for the entire excavation time, then there might be a considerable increase of the snow mass shifted per unit time. The reason for the higher effectiveness of DC could be that the clearing motion does not require the snow to be lifted. With clearing, the shearing resistance of snow has to be overcome. In our experiments, barely binding snow with low shearing resistance was used which lowered the excavation effort. Accordingly, the total excavation effort is expected to be lower with DC than DS for barely binding snow.

Men removed significantly more (17%) snow volume per scoop than women and the mean shoveling rate was significantly higher (25%) for men than for women. Therefore, the snow mass shifted per unit time was significantly (44%) higher for men than for women. This is most likely due to the different physical performance of men and women [19,20,21]. However, the physical performance of the tested women and men varied strongly. Some women even shifted more snow mass per unit time than some men. Therefore, every person involved in the rescue operation—regardless of sex—should be suitably deployed depending on physical performance.

Since the set task was clearing the box as quickly as possible, the participants started with maximum intensity. The maximum shoveling rate, which was reached in the first 20 s, was 67% higher for men and 91% higher for women than the mean shoveling rate. This is an indication of the high fatigue during the excavation over 3 to 5 min. To maximize snow transport, rescuers should be rapidly rotated (about 20–40 s). Accordingly, each rescuer could dig at high intensity without becoming exhausted. The alternating digging interval has to be increased if the rescuer change requires extra time. In steep terrain, when applying the V-shaped snow conveyor belt, the alternating of rescuers might be quite time consuming. The alternating time interval of four minutes, recommended by Genswein and Eide [12], should be reduced for short alternating times.

### Limitations

In several test runs, barely binding snow was the only snow type that provided reliable excavation results. This snow type is sometimes found in slab avalanches. The density of this snow with 651 kg/m^3^ is about equal to a springtime wet snow avalanche with 600 kg/m^3^ [22]. Stronger binding and more compressed snow is usually found in avalanches, which alters the procedure of excavations: cutting through the snow and breaking it up, as well as a controlled removal motion is required. Cutting and breaking the snow were not investigated in this study. It is noted that the shovel in the clearing mode might be inefficient for these activities. Snow partly slipped from the shovel due to the weak bonds between the snow grains. Wet or stronger binding snow may lead to different results since more snow can be loaded and less snow slips from the shovel. In another study [23], an attempt was made to create real avalanche snow conditions by shoveling snow approximately 25 m downhill and piling it up against a wooden wall.

The effectiveness of DC could not be determined due to the alternating from DC to DS during the excavation procedure. Moreover, for snow mass shifted per unit time and snow volume per scoop, only mean values over the whole excavation process could be determined.

To investigate the effectiveness of different shovel shapes in steep terrain, a different test set-up has to be developed. In steeper terrain, Genswein and Eide [12] recommend paddling motions so that the snow barely needs to be lifted; it can be pulled away. One can assume that a shovel used in clearing mode is more effective in steep terrain, as long as the snow does not need to be cut out.

If two or more rescuers are available, we recommend short alternating intervals due to the decrease in the shoveling rate. However, no experiments with exchanged rescuers were carried out to validate this recommendation.

## 5. Conclusions

A standardized laboratory test to evaluate the excavation effectiveness of avalanche shovels was developed. With a deep shovel blade with a straight shaft (DS), snow was moved faster than with a flat one (FS). The subsequent use of DC&DS (deep blade in clearing mode and deep blade with a straight shaft) proved to be most effective. Therefore, on avalanches, deep shovels should be used and, whenever possible, shovels with and without clearing mode should be alternated. Since the shoveling rate decreases after 20 s, rescuers have to be exchanged in short intervals, if no extra time for rescuer change is required and two or more rescuers are available. For this, a well-organized strategy is required. The different physical performance of every rescuer, especially of men and women, has to be taken into account when excavating a buried subject. Even though the test reliability made use of low bonding snow grains necessary, the results may be helpful to choose the right shovel type and the right strategy for efficiently excavating buried persons.

## Figures and Tables

**Table 1:** Snow mass shifted per unit time and snow volume per scoop. Flat shovel with a straight shaft (FS), deep shovel with a straight shaft (DS), and the subsequent use of two deep shovels with the shaft connected in clearing mode and straight mode (DC&DS). Values are given as mean ± SD for 14 women, 15 men and all participants. The increase between the shovel shapes is given in percent.

Shovel Shape	Snow Mass Shifted Per Unit Time (kg/s)			Snow Volume Per Scoop (cm^3^)		
	Women	Men	All Subjects	Women	Men	All Subjects
FS	1.23 ± 0.16	1.76 ± 0.30	1.50 ± 0.36	3073 ± 463	3551 ± 373	3330 ± 486
Differences FS-DS	14% *	14% *	14% *	16% *	16% *	15% *
DS	1.40 ± 0.15	2.01 ± 0.31	1.71 ± 0.39	3551 ± 486	4107 ± 414	3840 ± 521
Differences DS-DC&DS	4%	4%	4% *	3%	8% *	6% *
DC&DS	1.46 ± 0.15	2.10 ± 0.38	1.79 ± 0.43	3672 ± 377	4442 ± 517	4071 ± 580
Differences FS-DC&DS	18% *	19%*	19% *	19% *	25%*	22% *

* indicate statistically significant differences. The level of significance was set at *p* < 0.05.

**Table 2:** Mean and maximum shoveling rates. Flat shovel with a straight shaft (FS), deep shovel with a straight shaft (DS), and the subsequent use of two deep shovels with the shaft connected in clearing mode and straight mode (DC&DS). Values are given as mean ± SD for two groups: men and women. No significant differences between the shovel shapes were found. The level of significance was set at *p* < 0.05.

**Shovel Shape**	**Mean Shoveling Rate**			**Maximum Shoveling Rate**		
	**(Number of Scoops/min)**			**(Number of Scoops/min)**		
	**Women**	**Men**	**Difference**	**Women**	**Men**	**Difference**
FS	37.6 ± 4.9	47.6 ± 6.9	26.6% *	70.7 ± 9.2	78.0 ± 12.1	10.3% *
DS	36.9 ± 5.4	46.6 ± 6.3	26.3% *	69.0 ± 9.7	77.6 ± 11.4	12.5% *
DC&DS	37.1 ± 5	45.0 ± 6.7	21.3% *	73.3 ± 8.5	76.8 ± 12.5	4.8%

* indicate statistically significant differences. The level of significance was set at *p* < 0.05.

## Supplementary Files

Supplementary File 1

Supplementary File 2

Supplementary File 3

Supplementary File 4

Supplementary File 5

Supplementary File 6
